# Increased incidence of cytomegalovirus coinfection in HCV-infected patients with late liver fibrosis is associated with dysregulation of JAK-STAT pathway

**DOI:** 10.1038/s41598-017-10604-7

**Published:** 2017-09-04

**Authors:** Marwa K. Ibrahim, Ahmed Khedr, Noha G. Bader El Din, Ahmed Khairy, Mostafa K. El Awady

**Affiliations:** 1 Department of Microbial Biotechnology, Genetic Engineering Division, National Research Centre, 33 EL Bohouth St.(former El Tahrir St.), Dokki, Giza, P.O. 12622 Egypt; 2 Endemic Medicine Department, Faculty of Medicine, Cairo University, Giza, Egypt

## Abstract

Herein, we examined the association between cytomegalovirus (CMV) coinfection and the progression of liver fibrosis in hepatitis C virus (HCV) infection, and investigated the effect of CMV coinfection on JAK-STAT pathway. CMV DNAemia was detected by PCR in DNA from controls (n = 120), and HCV patients with early (F0-F1, n = 131) and late (F2-F4, n = 179) liver fibrosis. By quantitative real time PCR (qRT-PCR), we examined the profile of 8 JAK-STAT transcripts in PBMCs RNA from 90 HCV patients (39 CMV positive and 51 CMV negative), 4 CMV mono-infected patients, and 15 controls. Our results demonstrated higher incidence of CMV in F2-F4 group than in control (OR 5.479, 95% CI 3.033–9.895, p < 0.0001) or F0-F1 groups (OR 2, 95% CI 1.238–3.181, p = 0.005). qRT-PCR showed downregulation of STAT2 (p = 0.006) and IRF7 (p = 0.02) in CMV positive group compared to CMV negative one. The downregulation of STAT2 and IRF7 was mainly in CMV positive patients with late fibrosis compared to CMV negative patients (p = 0.0007 for IRF7 and p = 0.01 for STAT2). Our results are the first to report that CMV coinfection is a possible risk factor for the progression of HCV-induced liver fibrosis, and thereby CMV screening and treatment are important for HCV patients.

## Introduction

Hepatitis C virus (HCV) infection is a significant public health problem that affects as many as 170 million cases worldwide^1^. HCV targets either hepatocytes or extra-hepatic compartments such as peripheral blood mononuclear cells (PBMCs)^2^. Liver injury is the most serious clinical presentation of chronic HCV infection. It commences with liver inflammation and ultimately progresses to fibrosis, cirrhosis, and hepatocellular carcinoma (HCC) in the majority of patients. Despite of the present revolution in HCV therapies with much improvement in sustained virological response (about 68–74%)^3^, most of the patients are still at the risk of disease progression to cirrhosis and HCC at different rates.

Various etiological factors interplay to regulate the progression of hepatic fibrosis in HCV infection, including viral and host genetic factors^4^. Recently, increasing attention is given to coinfection as an underlying determinant for the progression of HCV-mediated liver diseases. Several studies showed that HCV/HIV and HCV/HBV coinfections lead to highly progressive liver diseases and poor response to IFN therapy^5^. The magnified pathophysiological influence of coinfection is thought to arise through increasing HCV replication, and/or provoking the immunosuppression effect.

Human cytomegalovirus (CMV) infects different body cells, including fibroblasts, endothelial and neuronal cells, and hepatocytes^6, 7^ besides blood monocytes and tissue resident macrophages (which both help in disseminating the infection throughout the body or serve as sites for latent infection)^8^. Although CMV causes subclinical latent infection in immunocompetent individuals, it causes severe symptoms in immunocompromised individuals^9^. Multiple reports elaborated the implication of CMV coinfection in the incidence and development of HCC^10, 11^ and in accelerating the progression rates of hepatic fibrosis after liver transplantation^12–14^.

Type I interferons (IFNα/β) are the major innate immunity mediators to battle against HCV infection. They are ultimately induced upon the recognition of HCV single stranded RNA (ssRNA) by host pattern recognition receptors. Once IFNα/β bind to their common heterodimeric receptor (IFNAR1/IFNAR2), they stimulate the signaling cascade of JAK-STAT pathway, mediating by the activation of JAK1 and TYK2, and ending in the formation of ISGF3 complex (IRF9, STAT1, and STAT2). The latter plays a key role in regulating the transcription of IFN-stimulated genes (ISGs), with the consequent killing of virus-infected cells and restricting virus replication^15^. Among these ISGs is IRF7, which works through a feed-back mechanism to induce the mRNA expression of a second wave of IFNα/β. By doing that, it allows type I IFN to create an antiviral state in neighboring cells^16^.

Multiple lines of investigations have shown that one of the most prominent immune evasion strategies of CMV is to interfere with JAK-STAT transduction in infected cells. CMV infected cells exhibit a decrement in STAT1 phosphorylation and translocation to the nucleus^17, 18^ and a decrease in IRF9 expression^19^. Additionally, CMV inhibits STAT2-dependent gene expression^20^.

There is a paucity of information regarding the coexistence of CMV and HCV infection, in particular its impact on the progression of liver diseases. We have shown in our previous reports higher incidence of CMV among HCV genotype 4 infected patients with less response to IFN therapy^21^, and treatment naïve patients having HCC^22^. To this end, in the present study we sought to investigate the frequency of CMV existence in HCV-infected patients with different grades of liver fibrosis. We further assessed the transcriptional profiling of genes involved in IFNα/β downstream pathway (JAK-STAT pathway) in PBMCs derived from those patients.

## Materials and Methods

### Ethical statement

All experiments were approved by the institution ethical review board (medical research ethics committee at National Research Center, Cairo, Egypt) according to Helsinki Declaration 1975 revised in 2008 and performed with the understanding of the human subject. Written informed consent was taken from each subject before blood collection and the ethics committee/institutional review board has approved the consent procedure.

### HCV-chronically infected patients

This study was conducted on 310 treatment-naïve HCV-chronically infected patients (genotype 4) having different degrees of liver fibrosis (early fibrosis (F0-F1, n = 131) and late fibrosis (F2-F4, n = 179)). The study subjects were recruited from Kasr Al-Aini, Endemic Medicine Department, Faculty of Medicine, Cairo University; and Viral Hepatitis Center, Ahmed Maher Teaching Hospital. The enrolled patients were HCV positive (seropositive and having detectable level of HCV-RNA in serum) and did not have any of the following: HBV surface antigen (HBsAg), markers for autoimmune diseases, antibodies for Schistosoma, uncontrolled type II diabetes mellitus, or any other etiologies causing chronic liver diseases. All the patients had no history of alcohol addiction and drug abuse. The degree of hepatic fibrosis was assessed histologically in liver biopsies by Metavir scoring system and confirmed by transient elastography (fibroscan) measurement.

### Healthy subjects

The enrolled 120 healthy subjects had no history of HCV infection (seronegative and having undetectable HCV-RNA in serum), HBV infection (negative HBsAg), Schistosoma infection, or autoimmune markers besides they had normal liver enzymes.

## CMV experiments

### DNA extraction

DNA was extracted from 200 µl whole blood collected on EDTA-coated tubes following the manufacturer’s instructions of Qiagen DNA extraction kit (Qiagen, Santa Clarita, CA).

### Amplification of CMV DNA

CMV DNA was detected in PBMCs by nested PCR amplification using specific primers for the CMV *gB* region as described before^23, 24^. Both PCR rounds had similar thermal cycling protocol, which started with initial denaturation at 94 °C for 5 min then 35 cycles of 1 min at 94 °C, 1 min at 55 °C, and 1 min at 72 °C, and ended with final extension at 72 °C for 10 min. The 100 bp nested amplicon was electrophoresed on agarose gel (3%) stained with ethidium bromide.

### Detection of CMV immunoglobulin

CMV-specific IgG and IgM were detected in serum by enzyme-linked immunosorbent assay (ELISA) kit (DRG international, Inc, New Jersy, USA) according to the manufacturer’s instructions. The samples were measured at OD 450 nm using ELISA reader (TECAN; SUNRISE, Austria, GmbH).

## Gene expression experiments

### RNA extraction

RNA was extracted from 3 ml freshly drawn blood samples following the protocol of the single-step method^25^. The recovered RNA was quantified using Thermo Scientific NanoDrop™ Spectrophotometer.

### qRT-PCR analysis

250 ng of total cellular RNA was reverse transcribed into cDNA using RT^2^ PCR First Strand Kit (SABiosciences, Valencia, CA). For qRT-PCR assay, a reaction mix conatining 12.5 µl RT^2^ SYBR Green/ROX qPCR master mix (SABiosciences), 10.5 µl nuclease free water, 1 µl of cDNA, and 1 µl of gene-specific PCR primer for human *IFNAR1, IFNAR2, STAT1, STAT2, JAK1, TYK2, IRF9*, or *IRF7* (10 µM; SABiosciences) was prepared and analyzed on Rotor Gene real-time PCR system (Qiagen). The house keeping gene human *B2M* (SABiosciences) was used in a separate tube for normalization. The thermal cycling protocol started with initial incubation at 95 °C for 10 min (AmpliTaq Gold pre-activation), followed by 40 cycles at 95 °C for 15 sec and 60 °C for 1 min. Relative mRNA expression of each gene was estimated by the 2^−ΔΔCT^ method and presented as fold change compared to the mean of the control group.

### Statistical analysis

The Prism software version 5 (GraphPad, La Jolla, CA) and IBM SPSS statistics software version 16 were used to perform the statistical analyses described in the study. The clinical parameters and gene expression data were analyzed using either the parametric unpaired t test or the non-parametric Mann-Whitney U test (according to following the normal distribution curve), and presented as mean and standard error of the mean. The frequency of CMV coinfection within different groups was analyzed by χ2 test, Fisher exact, and binary logistic regression analysis; and described as odds ratio with 95% confidence interval (CI). Difference between groups was considered statistically significant if p ≤ 0.05.

### Data Availability

All data generated or analysed during this study are included in this published article.

## Results

### Description of the study patients

Table 1 summarizes demographic and biochemical parameters for all the study patients (n = 310). Early and late fibrosis patients showed significant variations in a few of the measured indices, including BMI, total bilirubin, and platelets count (Table 1, p = 0.01, 0.02, and < 0.0001; respectively). Histopathological examination of the liver revealed higher degrees of both steatosis and liver inflammation among patients with late fibrosis than those with early fibrosis (p = 0.0002 and < 0.0001; respectively, Table 1). However, the other variables (gender, age, albumin, HB, ALT, and AST; Table 1) were similar in both groups of patients. Table 2 shows the clinical characteristics of the study patients (n = 90) chosen for the qRT-PCR experiment; performed to measure mRNA abundance of JAK-STAT pathway-related transcripts. Early and late fibrotic patients showed significant difference in age (Table 2, p = 0.01) and comparable levels of the other indices (gender, BMI, total bilirubin, albumin, HB, ALT, and AST, and platelets count; Table 2). Data on steatosis and liver activity were not available for the majority of the 90 patients.

**Table 1:** Clinical features of the 310 HCV- infected patients with early and late stages of liver fibrosis; selected for studying CMV prevalence.

	Early fibrosis (F0-F1, n = 131)	Late fibrosis (F2-F4, n = 179)	*P* value
Female/Male	87/44	100/79	NS
Age (years)	40.5 ± 0.9	42.6 ± 0.9	NS
BMI (kg/m2)	25 ± 0.5	27 ± 0.6	0.01
HCV viral load (IU/mL)	876,310 ± 209,946	1,035,088 ± 259,046	NS
Bilirubin total (mg/dL)	0.7 ± 0.04	0.9 ± 0.05	0.02
Albumin (g/dL)	3.6 ± 0.1	3.5 ± 0.08	NS
HB (g/dL)	11.9 ± 0.2	11.7 ± 0.21	NS
ALT (U/L)	42.9 ± 3	42 ± 3.1	NS
AST (U/L)	45 ± 3.4	46.8 ± 3.**7**	NS
Platelets count (cmm3)	259,000 ± 9,200	201,000 ± 7,700	<0.0001
^**1**^ **Steatosis**			
<33%	50 (77%)	38 (43%)	0.0002
33–66%	10 (15%)	33 (37.5%)	
>66%	5 (8%)	17 (19.5%)	
^**2**^ **Liver activity**			
A1	39 (91%)	20 (28%)	<0.0001
A2	4 (9%)	46 (64%)	
A3	0	6 (8%)	

n indicates to the sample size. BMI refers to body mass index, HB refers to hemoglobin, ALT refers to serum alanine aminotransferase, and AST refers to serum aspartate aminotransferase. ^1^Degree of steatosis was estimated in 153 patients (65 F0-F1 and 88 F2-F4). ^2^Liver activity was detected in 115 patients (43 F0-F1 and 72 F2-F4). Data are expressed as mean and standard error except for steatosis and liver activity, data are expressed as total number and percent. NS refers to non-significant p value (p > 0.05).

**Table 2:** Clinical features of the 90 HCV- infected patients with early and late hepatic fibrosis; selected for studying the transcriptional profile of JAK-STAT pathway mediators.

	Early fibrosis (F0-F1, n = 39)	Late fibrosis (F2-F4, n = 51)	*P* value
Female/Male	30/9	38/13	NS
Age (years)	36.2 ± 2.4	44.8 ± 2.3	0.01
BMI (kg/m2)	27.5 ± 0.6	27 ± 3.3	NS
Bilirubin total (mg/dL)	0.6 ± 0.09	0.7 ± 0.09	NS
Albumin (g/dL)	4.07 ± 0.1	3.9 ± 0.08	NS
HB (g/dL)	13.1 ± 0.41	12.5 ± 0.35	NS
ALT (U/L)	26.7 ± 4.7	37 ± 5.3	NS
AST (U/L)	27.8 ± 4.9	36 ± 4.9	NS
Platelets count (cmm3)	278,900 ± 18,300	237,800 ± 13,400	NS

Data are expressed as mean and standard error of mean. n indicates to the sample size. BMI refers to body mass index, HB refers to hemoglobin, ALT refers to serum alanine aminotransferase, and AST refers to serum aspartate aminotransferase. NS refers to non-significant p value (p > 0.05).

### Prevalence of CMV antibodies in HCV-chronically infected patients

Table 3 reveals that all the study patients had a past exposure to CMV as evident by the presence of detectable levels of CMV-specific IgG in their sera. Reactivated latent infection or primary infection (based on CMV DNA positivity) was observed in 41% of the patients (n = 126/310, Table 3). From those, active primary infection (based on IgM positivity) was observed in 19% of the patients (n = 24, Table 3); and reactivated latent infection (based on IgM negativity and IgG positivity) was observed in 81% of the patients (n = 102). 5 HCV patients showed CMV IgM and IgG positivity, but with CMV DNA under the detectable levels (Table 3).

**Table 3:** Comparison between serum levels of IgG and frequency of IgM positivity in CMV DNAemia positive and negative patients.

	CMV DNAemia positive patients	CMV DNAemia negative patients	*P value*
CMV DNA	126/310 (41%)	184/310 (59%)	
CMV IgG (IU/mL)	12.8 ± 1.7	12.5 ± 1.3	NS
CMV IgM	24/126 (19%)	5/184 (3%)	< 0.0001

IgG titer is expressed as mean and standard error of mean. Cut off for IgM positivity is 0.7863 OD. NS refers to non-significant p value (p > 0.05).

### Frequency of CMV coinfection among HCV-chronically infected patients having different grades of liver injuries

**Figure 1:**
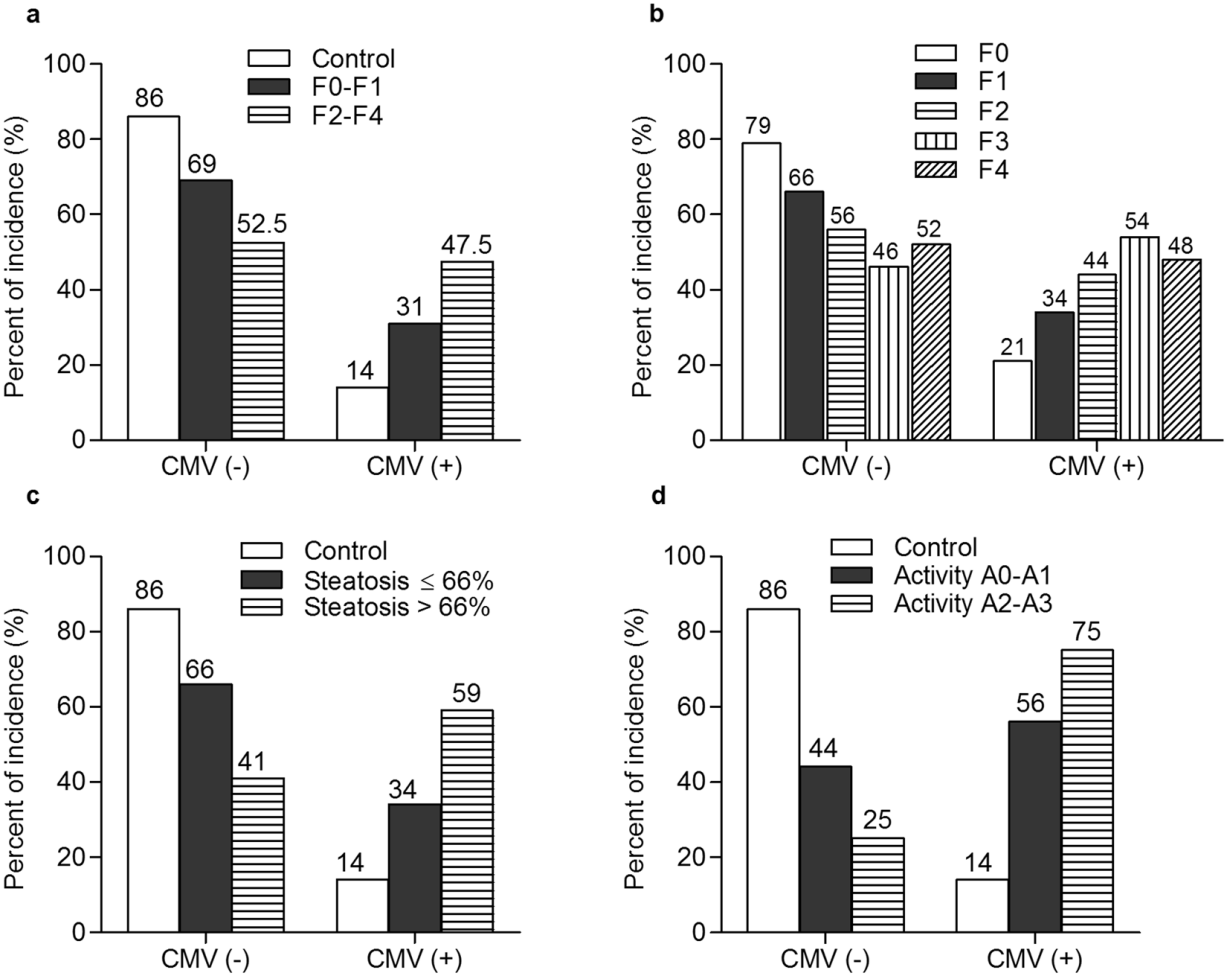
The frequency of CMV infection in healthy subjects and HCV patients with different grades of liver pathologies. The percent of CMV DNAemia positivity was determined in healthy controls (n = 120) and HCV-patients with different stages of liver pathologies. (**a**) HCV-patients with early (F0-F1, n = 131) and late (F2-F4, n = 179) liver fibrosis. (**b**) HCV-patients with individual stage of liver fibrosis, (F0 (n = 29), F1(n = 102), F2 (n = 78), F3 (n = 37), and F4 (n = 64)). (**c**) HCV-patients with different grades of steatosis (≤66% (n = 131) and >66% (n = 22)). (**d**) HCV-patients with different grades of hepatic activity (A0-A1 (n = 59) and A2-A3 (n = 56)).

To gain perspectives on the influence of CMV coinfection on the clinical outcome of liver pathologies, blood samples collected from healthy subjects and HCV-patients with different grades of liver fibrosis, steatosis, and hepatitis activity were screened for CMV DNA. Based on IgM and IgG data, we attributed CMV DNAemia positivity detected in 81% of the patients to the reactivation of CMV latent infection (IgG positive and IgM negative, n = 102), and in 19% of the patients to the primary CMV infection (IgM positive, n = 24). As illustrated in Table 4, CMV DNAemia was significantly higher in HCV infected-patients than in healthy subjects (p < 0.0001 (OR 4.149, 95% CI 2.367–7.271)). Strikingly, the incidence of CMV was more frequent in the late fibrosis group than in the early one (Table 4 and Fig. 1a, p = 0.005). The prevalence of CMV coinfection showed a stepwise increase as the severity of liver fibrosis goes up (Fig. 1b, CMV DNAemia 21% in F0 (total sample size = 29), 34% in F1 (total sample size = 102), 44% in F2 (total sample size = 78), 54% in F3 (total sample size = 37), and 48% in F4 (total sample size = 64)). Interestingly, CMV coinfection exhibited a profound effect on the other aspects of liver pathologies. CMV coinfection was more frequent in HCV patients with higher grades of both hepatic steatosis ( > 66%, p = 0.046; Table 4 and Fig. 1c) and hepatitis activity (A2–A3, p = 0.04; Table 4 and Fig. 1d). Binary logistic regression analysis showed that the probability of the progression of liver fibrosis to late stages (F2-F4) is higher in HCV patients with positive CMV DNAemia (Table 5, p = 0.004 (OR 2 95% CI 1.239–3.181)). Moreover, HCV/CMV co-infected patients showed increased risk of steatosis progression ( > 66%) and liver inflammation (A2-A3) than HCV mono-infected patients (Table 5; p = 0.03 (OR 2.8 95% CI 1.133–7.197) for steatosis and p = 0.04 (OR 2.4 95% CI 1.064–5.295) for liver inflammation). Collectively, these results present CMV coinfection as a possible risk factor for the progression of HCV-associated liver injuries.

**Table 4:** Frequency of CMV coinfection in healthy controls and HCV- infected patients having different grades of liver diseases.

	CMV DNAemia negative n (%)	CMV DNAemia positive n (%)	*P value*
Healthy subjects (n = 120)	103 (86)	17 (14)	<0.0001
All HCV patients (n = 310)	184 (59)	126 (41)	
**Fibrosis**			
F0-F1 (n = 131)	90 (69)	41 (31)	0.005
F2-F4 (n = 179)	94 (52.5)	85 (47.5)	
**Steatosis**			
< 33% (n = 88)	56 (64)	32 (36)	0.046
33–66% (n = 43)	31 (72)	12 (28)	
> 66% (n = 22)	9 (41)	13 (59)	
**Liver activity**			
A0-A1 (n = 62)	27 (44)	35 (56)	0.04
A2 (n = 47)	13 (28)	34 (72)	
A3 (n = 6)	0 (0)	6 (100)	

n is the sample size. F0-F1 refers to early fibrosis. F2-F4 refers to late fibrosis.

**Table 5:** Binary logistic regression analysis for the impact of CMV coinfection on the progression of liver diseases.

CMV DNAemia positive vs CMV DNAemia negative	Regression coefficient	SE	OR (95% CI)	*P* value
Fibrosis (F0-F1 vs F2-F4)	0.686	0.241	2 (1.239–3.181)	0.004
Steatosis (≤60% vs >60%)	1.049	0.471	2.8 (1.133–7.197)	0.03
Activity (A0-A1 vs A2-A3)	0.864	0.407	2.4 (1.064–5.295)	0.04

SE, standard error; OR, odds ratio; CI, confidence interval.

### Increased incidence of CMV coinfection among HCV chronically infected patients is associated with dysregulation of JAK-STAT pathway

**Figure 2:**
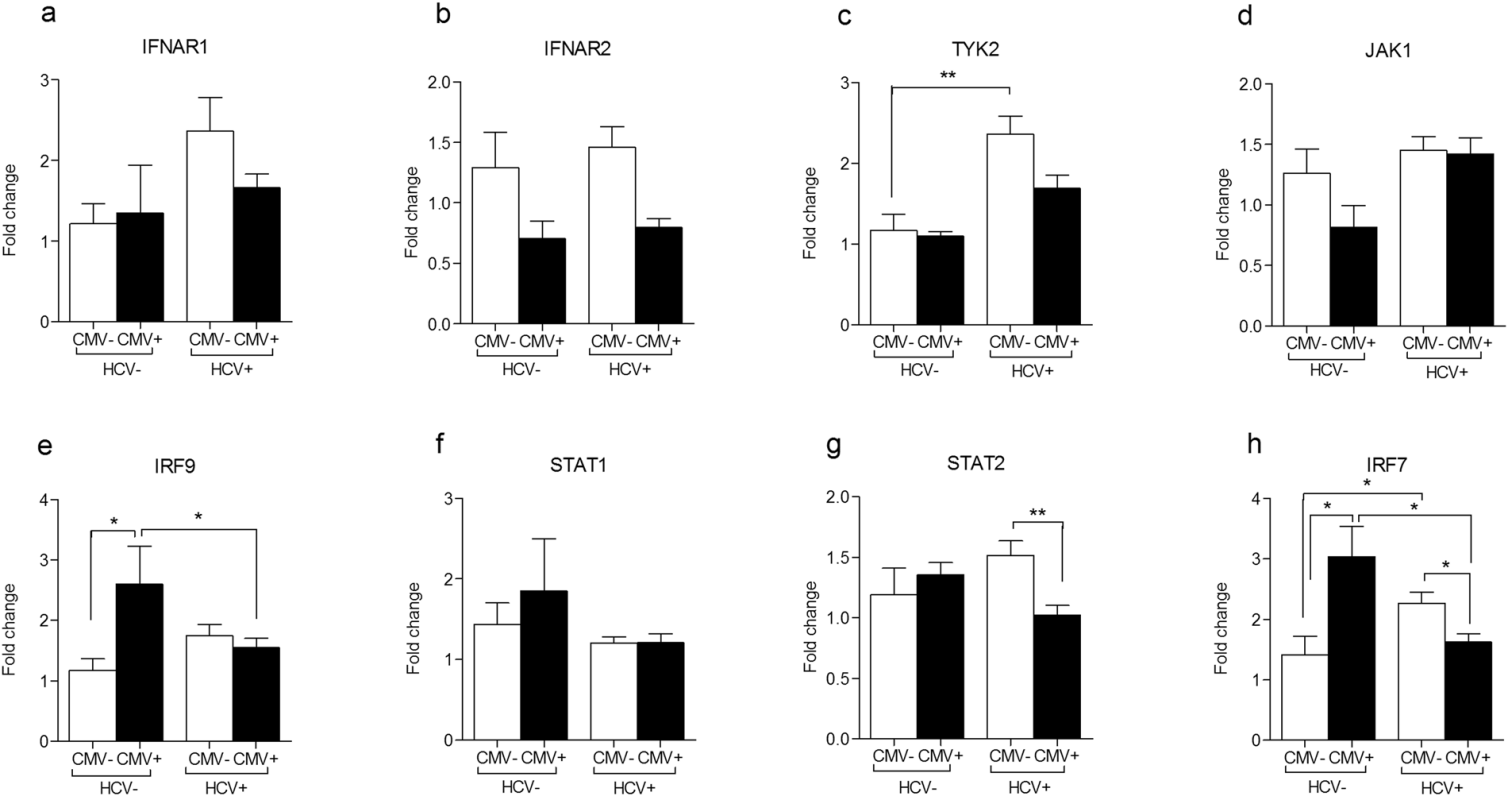
The transcriptional levels of JAK-STAT pathway mediators in CMV positive versus CMV negative chronic HCV-infected patients. qRT-PCR was employed to quantify the mRNA abundance of IFNAR1 (**a**), IFNAR2 (**b**), TYK2 (**c**), JAK1 (**d**), IRF9 (**e**), STAT1 (**f**), STAT2 (**g**), and IRF7 (**h**) in PBMCs of control group (CMV− HCV−, n = 15), CMV mono-infected patients (CMV+ HCV−, n = 4), and HCV-infected patients with positive CMV DNAemia (HCV+ CMV+ , n = 39) and with negative CMV DNAemia (HCV+ CMV−, n = 51). The house keeping gene B2M was used for data normalization. The relative gene expression of each transcript was presented as fold change comparative to the mean of control group. (*p < 0.05; **p < 0.01).

It is well known that CMV interferes with JAK-STAT signaling pathway. Therefore, we sought next to investigate the impact of CMV coinfection on the regulation of the main antiviral innate immunity pathway i.e., JAK-STAT pathway. We used qRT-PCR to analyze the expression profile of 7 key transcripts in IFNα/β downstream signaling pathway; IFNAR1, IFNAR2, STAT1, STAT2, JAK1, TYK2, and IRF9, besides one ISG (IRF7); which plays a key role in regulating this pathway. The expression level of the aforementioned transcripts was measured in PBMCs RNA from 90 treatment-naïve chronic HCV patients (39 CMV positive and 51 CMV negative), 4 CMV mono-infected patients (HCV negative), and 15 healthy subjects (CMV negative and HCV negative). HCV mono-infected patients were able to upregulate only TYK2 and IRF7 in response to the virus infection (Fig. 2c and h, p = 0.007 and 0.01; respectively), while CMV mon-infected patients showed only upregulation of IRF9 and IRF7 (Fig. 2e and h, p = 0.04 and 0.05; respectively). The expression level of rest of the 8 transcripts was comparable between the mono-infected groups and the healthy individuals. Most of the studied genes (IFNAR1, IFNAR2, STAT1, JAK1, TYK2, and IRF9) showed similar pattern of regulation between HCV patients with or without CMV DNAemia (Fig. 2, p > 0.05 for all). STAT2 showed less mRNA abundance in HCV/CMV co-infected patients upon comparing to HCV mono-infected group (Fig. 2g, p = 0.006). Although IRF7 showed upregulation in both HCV mono-infected patients and CMV mono-infected patients when compared to the healthy subjects, its transcriptional level was downregulated upon coinfection (Fig. 2h; p = 0.02 for both HCV mono-infected versus HCV/CMV patients and for CMV mono-infected versus HCV/CMV patients). Together, these data indicate that CMV coinfection alters the regulation of JAK-STAT pathway in HCV-chronically infected patients.

### Increased incidence of CMV coinfection among HCV infected patients with higher grades of liver fibrosis is associated with dysregulation of JAK-STAT pathway

**Figure 3:**
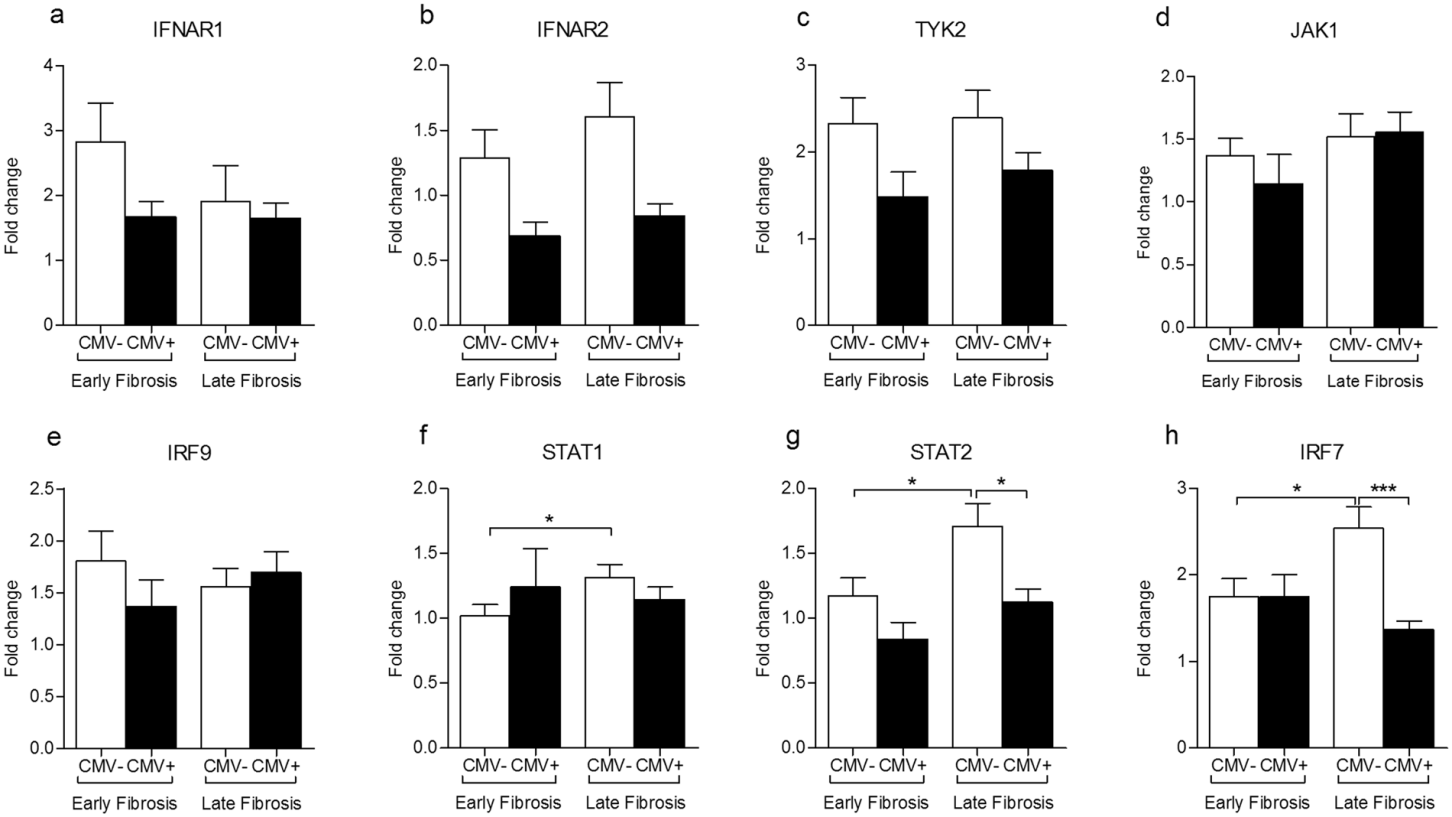
The transcriptional levels of JAK-STAT pathway mediators in CMV positive versus CMV negative chronic HCV-infected patients with different stages of liver fibrosis. qRT-PCR was employed to quantify the mRNA abundance of IFNAR1 (**a**), IFNAR2 (**b**), TYK2 (**c**), JAK1 (**d**), IRF9 (**e**), STAT1 (**f**), STAT2 (**g**), and IRF7 (**h**) in PBMCs of HCV mono-infected patients with early (F0-F1 (CMV−), n = 23) and late (F2-F4 (CMV−), n = 28) hepatic fibrosis, and HCV/CMV co-infected patients with early (F0-F1 (CMV+ ), n = 13) and late (F2-F4 (CMV+ ), n = 26) hepatic fibrosis. The house keeping gene B2M was used for data normalization. The relative gene expression of each transcript was presented as fold change comparative to the mean of control group. (*p < 0.05; ***p < 0.001).

Next, we tried to understand the mechanism underlying the accelerated progression of HCV-induced liver fibrosis in CMV DNAemia positive patients. To reach this goal, we further classified our HCV-patients into 4 groups based on CMV coinfection and severity of liver fibrosis; HCV mono-infected patients with early (F0-F1, n = 23) and late (F2-F4, n = 28) hepatic fibrosis, and HCV/CMV co-infected patients with early (F0-F1, n = 13) and late (F2-F4, n = 26) hepatic fibrosis. We assessed the expression levels of the above mentioned JAK-STAT pathway mediators (IFNAR1, IFNAR2, STAT1, STAT2, JAK1, TYK2, IRF9, and IRF7) among the 4 groups of patients. No significant differences were found in the expression levels of the 8 transcripts between HCV mono-infected and HCV/CMV co-infected patients with early grades of liver fibrosis (Fig. 3a–h). Regarding HCV patients with late fibrosis, CMV coinfection led to a dramatic down regulation in two transcripts, STAT2 and IRF7 (Fig. 3g,h, p = 0.01 and 0.007; respectively). Seemingly, in HCV mono-infection, STAT2 and IRF7 gene expression showed around 1.5 fold upregulation with the progression of liver fibrosis i.e. when the comparison was set between CMV negative patients with early fibrosis vs CMV negative patients with late fibrosis (Fig. 3g,h, p = 0.04 and 0.03 for STAT2 and IRF7; respectively). However, CMV coinfection interfered with the upregulation of the two transcripts when liver fibrosis moved from early to late stages (Fig. 3g,h). These data suggest that the increased severity of HCV-induced liver fibrosis in CMV coinfection is likely due to CMV-driven dysregulation of JAK-STAT pathway.

## Discussion

Understanding the key factors regulating the progression of liver fibrosis in HCV infection is a real challenge. Several studies presented coinfection as a master player in this context^26, 27^. We explored in our previous studies increased occurrence of CMV coinfection in HCV-patients responding poorly to IFN-based therapeutics^21^ and treatment-naïve patients with HCC^22^. The findings reported in the present study demonstrate that CMV coinfection profoundly impacts the severity of HCV-initiated liver diseases (liver inflammation, steatosis, and fibrosis). The noticeable increased incidence of CMV coinfection in patients with late fibrosis stage is associated with dysregulation of IFNα/β downstream pathway (JAK-STAT pathway) in their PBMCs. These data shed light on a new determinant for accelerated hepatic fibrosis in setting of HCV infection, and provide a foundation for understanding the underlying immunological events.

The progression of liver injuries associated with HCV chronicity is a multi-factorial process. In this regard, HCV co-infection with other viruses is reasoned to be one of the most important monitoring factors. Recent studies have reported that HCV patients co-infected with HBV or HIV have highly progressive liver diseases and rapidly reach cirrhosis and HCC than HCV mono-infected patients^26–28^. However, reports on the coexistence of CMV with HCV are scarce and mainly centering on the role of CMV after liver or kidney transplantation. The present study is the first to show an intimate relationship between CMV and the progression of liver injuries (liver inflammation, steatosis and fibrosis) from early to advanced grades in HCV genotype 4 infected patients. In accord with our results, several publications demonstrated augmented severity of hepatitis activity index and fibrosis status in HCV/CMV co-infected patients undergoing liver transplantation^12–14^. HBV and HCV patients co-infected with CMV suffer from severe necroinflammation and liver fibrosis than CMV mono-infected patients^29^. Patients with non-alcoholic fatty liver diseases have high prevalence of Herpesviridae family, including CMV^30^. Our earlier work showed a direct relationship between CMV coinfection and weak response to IFN therapy^21^, as well as increased risk of HCC development^22^. CMV establishes a lifelong latency in healthy individuals; however, upon immunosuppression, reactivation occurs due to re-initiation of the viral lytic replication program^31^. Chronic HCV infection represents an ideal example for immunosuppression milieu. Regulatory T cells exhibit increased numbers and enhanced suppressive activity in HCV-chronically infected patients versus spontaneous clearance^32–34^. On the contrary, natural killer cells and CD8^+^ T cells are present with reduced expression of activating cytotoxic receptors and elevated expression of inhibitory receptors in chronic HCV-infected patients^35^. Our data of increased incidence of CMV reactivation in HCV infected patients (regardless the liver pathologies), when compared to healthy subjects, present an evidence for the idea that HCV-patients are immunosuppressed.

The first reaction of host defense against viruses is through the secretion of IFNs; which start certain transcriptional programs to stimulate innate immunity and limit viral replication. The binding of type I IFNs (IFNα/β) to the heterodimeric receptor complex (IFNAR1–IFNAR2) activates receptor-associated kinases JAK1 and TYK2, and provokes tyrosine phosphorylation of STAT1 and STAT2^36^. The dimerized STAT1 and STAT2 recruits IRF9 and forms ISGF3, which binds to IFN-stimulated response elements and stimulates the expression of several immunity genes (ISGs)^36–38^. Several studies have shown the modulation of JAK/STAT signaling pathway by HCV infection. In cell lines transfected with HCV replicons, STAT1 phosphorylation was impaired due to both core and NS5A interaction^39, 40^. In another study, IRF7 and STAT1 showed compromised phosphorylation and nuclear translocation^41^. In hepatocytes derived from liver biopsies collected from chronic HCV patients, STAT1 phosphorylation was efficient, but the binding of STAT1 to the promoters of ISGs was affected due to STAT1 hypomethylation^42^. Although there is much focus on studying the modulatory role of HCV in STAT1 induction and function, the effect of HCV infection on the other mediators of JAK/STAT pathway is poorly studied. In our study we noticed similar mRNA expression of all the studied transcripts between HCV infected patients versus healthy controls except for TYK2 and IRF7, which showed upregulation. To our knowledge this study is the first to depict the transcriptional regulation of JAK-STAT pathway in HCV genotype 4.

CMV counteracts the host immunity by targeting type I IFN signaling pathway at several steps: (1) CMV blocks IFNAR1, STAT2, STAT1, and TYK2 phosphorylation, as well as STAT1 nuclear translocation^18, 43^, (2) CMV lessens the levels of JAK1 protein by enhancing the proteasomal degradation, although JAK1 mRNA level remains constant^43^; which is consistent to our finding, (3) CMV IE1 protein pp72 forms a complex with STAT2, and thereby inhibits STAT2-induced gene expression^20^, and (4) CMV induces STAT2 degradation^44^. In the current study, we report another strategy for CMV to interfere with type I IFN signaling pathway through downregulation the mRNA expression of STAT2. Discordant with our study, Le *et al*.^44^ found augmented abundance of STAT2 mRNA in MRC-5 cells infected with human CMV strain when compared to mock infected cells, suggesting differential response of different cell types (fibroblasts in Le study versus PBMCs in our study) to human CMV regarding STAT2 mRNA regulation, and the possible contribution of different human CMV strains to this context. As a secondary effect for CMV-mediated inhibition of IFN pathway, the expression of ISGs is diminished in CMV infected cells. IFN-treated human fibroblasts stably expressing the CMV protein IE1–72kDa show low abundance of ISG54 and MxA mRNA^20^. In another report, both OAS and MxA show undetectable RNA level in CMV-infected fibroblasts and endothelial cells^17^. Supportive of the CMV-mediated dysregulation of JAK-STAT pathway observed in our study, mRNA expression of the ISG, IRF7, was also downregulated. The latter confirms that STAT-dependent gene expression is also impaired in HCV/CMV co-infected patients. IRF7 plays an additional role in regulating JAK-STAT pathway through inducing the transcription of IFNα/β. Therefore, IRF7 downregulation is likely to contribute to the overall dysregulation of JAK-STAT pathway through diminishing the mRNA expression of the pathway ligands (IFNα/β), which we already noticed in our previous work (unpublished data). The pronounced decrement in IRF7 expression in HCV/CMV co-infected patients either when compared to HCV or CMV mono-infected patients indicates that the dysregulation of JAK/STAT pathway is likely due to the interaction of CMV and HCV proteins.

As shown by several reports, HCV RNA can be transmitted from HCV bearing liver cells to plasmacytoid dendritic cells (pDCs), which respond promptly by producing IFN^45^. The trafficking of pDCs (the professional type I IFN-producing cells at levels 100-1000 times higher than that produced by any other blood cell type) from liver back to blood has a great impact on the gene expression of IFN related transcripts in PBMCs^46^. Accordingly, the levels of JAK-STAT mediators in PBMCs is likely to reflect the inflammatory status of the liver. Signaling through JAK–STAT pathway plays a protective role against liver fibrogenesis. For example, IFN-α inhibits the transcription of collagen gene, and thereby improves the degree of liver fibrosis in an experimental mouse model^47^. STAT1 induces hepatic stellate cells apoptosis and cell cycle arrest, and by doing that acts as an anti-fibrotic factor^48^. Although STAT2 is a well-known antiviral molecule, its role in liver injury and fibrosis is not examined yet. Our results clearly revealed a failure to upregulate STAT2 and IRF7 in HCV/CMV co-infected patients with high grades of liver fibrosis when compared to HCV mono-infected patients. Till now, nothing is known about the molecular mechanism underlying the CMV-related increased severity of liver diseases, and the panel of viral proteins implicated in this process. Indeed, the majority of the current studies are listed under the observational category. However, the previous reports on the pathogenicity of CMV in other tissues introduced some CMV proteins as inducers for the fibrogenesis process. Transfection of renal epithelial cells with plasmids encoding the human CMV IE1 or IE2 gene products showed their possible role in the fibrogenesis process. The latter conclusion is evident by the potency of IE1 and IE2 gene products in inducing TGF-β1 activation (the well-known potent fibrogenic molecule) with the consequent acquiring of the fibrogenic phenotype by the transfected cells^49^. Of more important, several CMV proteins modulate the cellular apoptotic machinery^50^. CMV UL97 protein inactivates the retinoblastoma tumor suppressor in mammalian cells^51^, CMV UL36 protein inhibits Fas-mediated apoptosis^52^, and CMV IE86 protein binds to p53 with the consequent inhibition of apoptosis^53^. Our study introduces the dysregulation of the anti-fibrotic pathway i.e., JAK/STAT pathway as a possible scenario for CMV-mediated increased severity of liver fibrosis in HCV infection. We showed in our previous report an increased incidence of CMV coinfection among HCV patients with HCC^22^. The dysregulation of JAK-STAT pathway in HCV/CMV co-infected patients with advanced stage of liver fibrosis is likely to be a key molecular and immunological factor for increased susceptibility to develop HCC. However, further longitudinal studies are needed to support the latter notion.

The significance of the current findings is limited by the absence of confirmatory experiments at translational and posttranslational levels, and lack of follow up experiments needed to investigate the consequences of CMV-mediated dysregulation of JAK-STAT pathway, and whether it accelerates the progression of liver fibrosis to HCC.

To our knowledge our study is the first to report on the role of CMV coinfection in the progression of HCV genotype 4-induced hepatic fibrosis from early to advanced stages. In conclusion, screening for CMV is of great importance among HCV patients. Treating CMV active infection using the available therapeutic interventions is very important to lessen the clinical outcome of HCV chronic infection. The precise mechanism underlying CMV and augmented severity of liver fibrosis needs to be determined. Our data on the dysregulation of JAK/STAT pathway present a foundation for future mechanistic studies.
